# Supplementary figures and images for: Whole-Blood Gene Expression Profiles Correlate with Response to Immune Checkpoint Inhibitors in Patients with Metastatic Renal Cell Carcinoma
Source: Cancers (Basel). 2022 Dec 15;14(24):6207. doi: 10.3390/cancers14246207 (PMC9776722; doi:10.3390/cancers14246207)

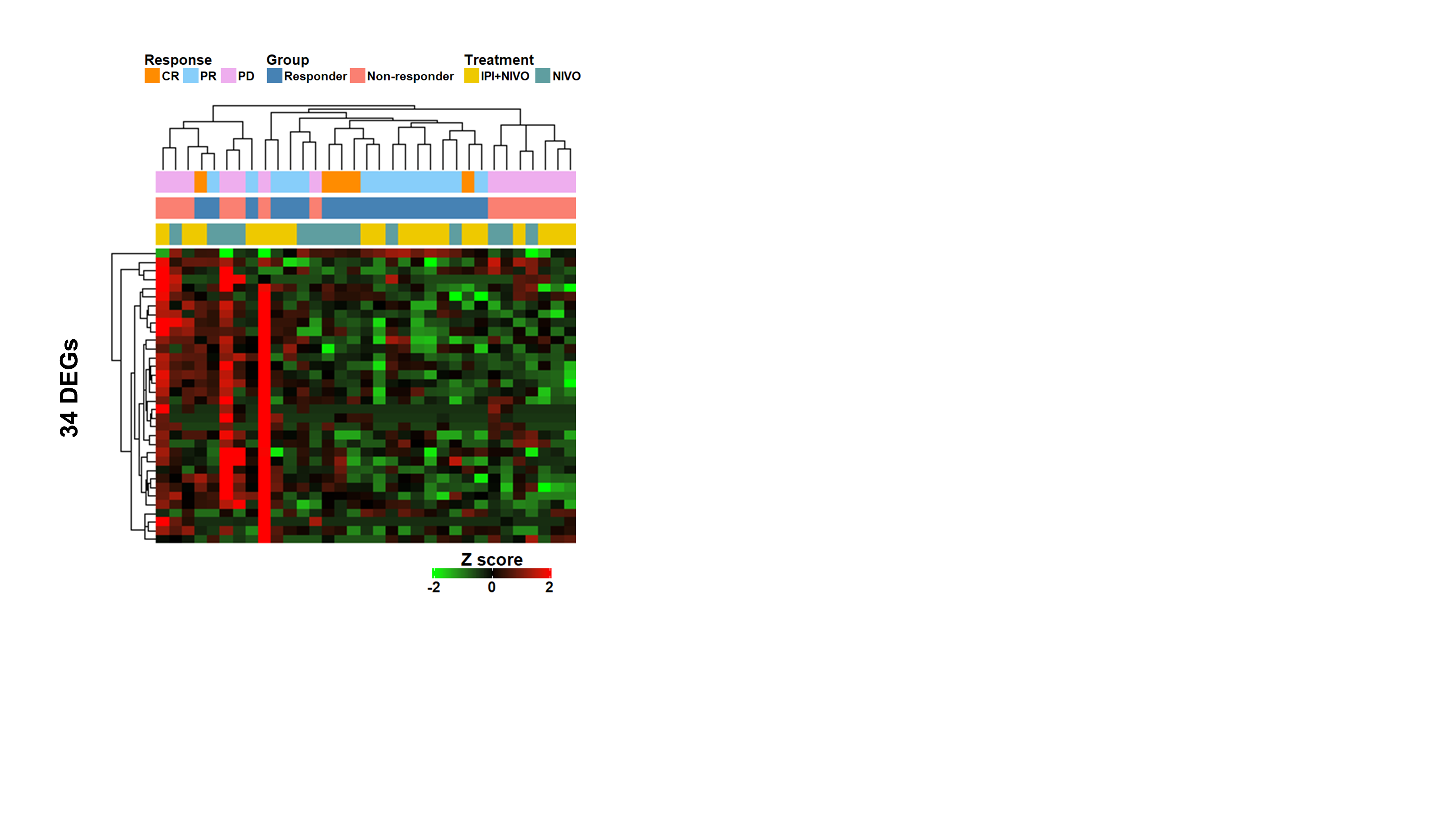

Supplement: Supplementary file 1 [file cancers-14-06207-s001.zip › Supplementary File_for_revision_20221215_v2/FigureS1.tif]

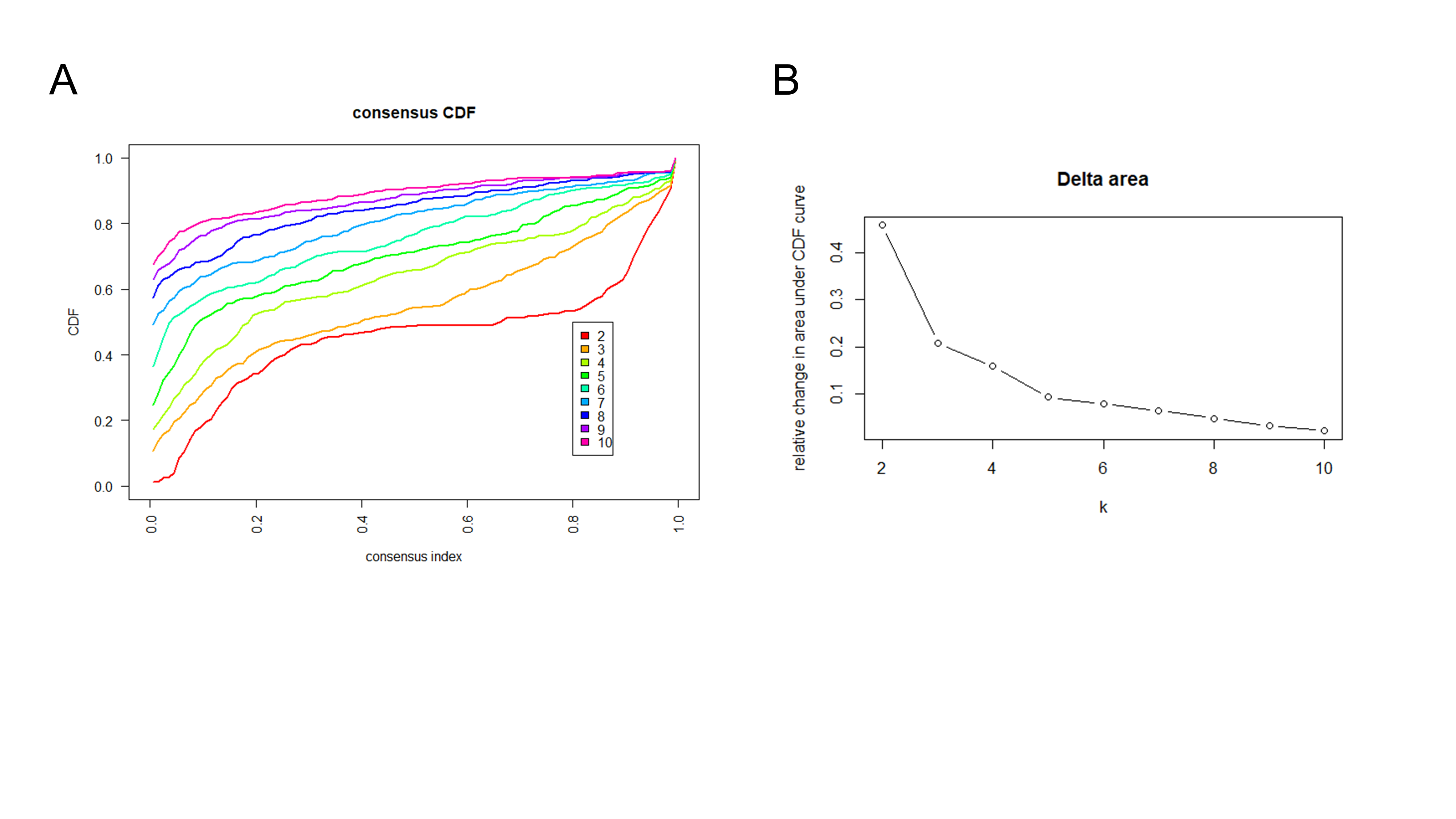

Supplement: Supplementary file 1 [file cancers-14-06207-s001.zip › Supplementary File_for_revision_20221215_v2/FigureS2.tif]

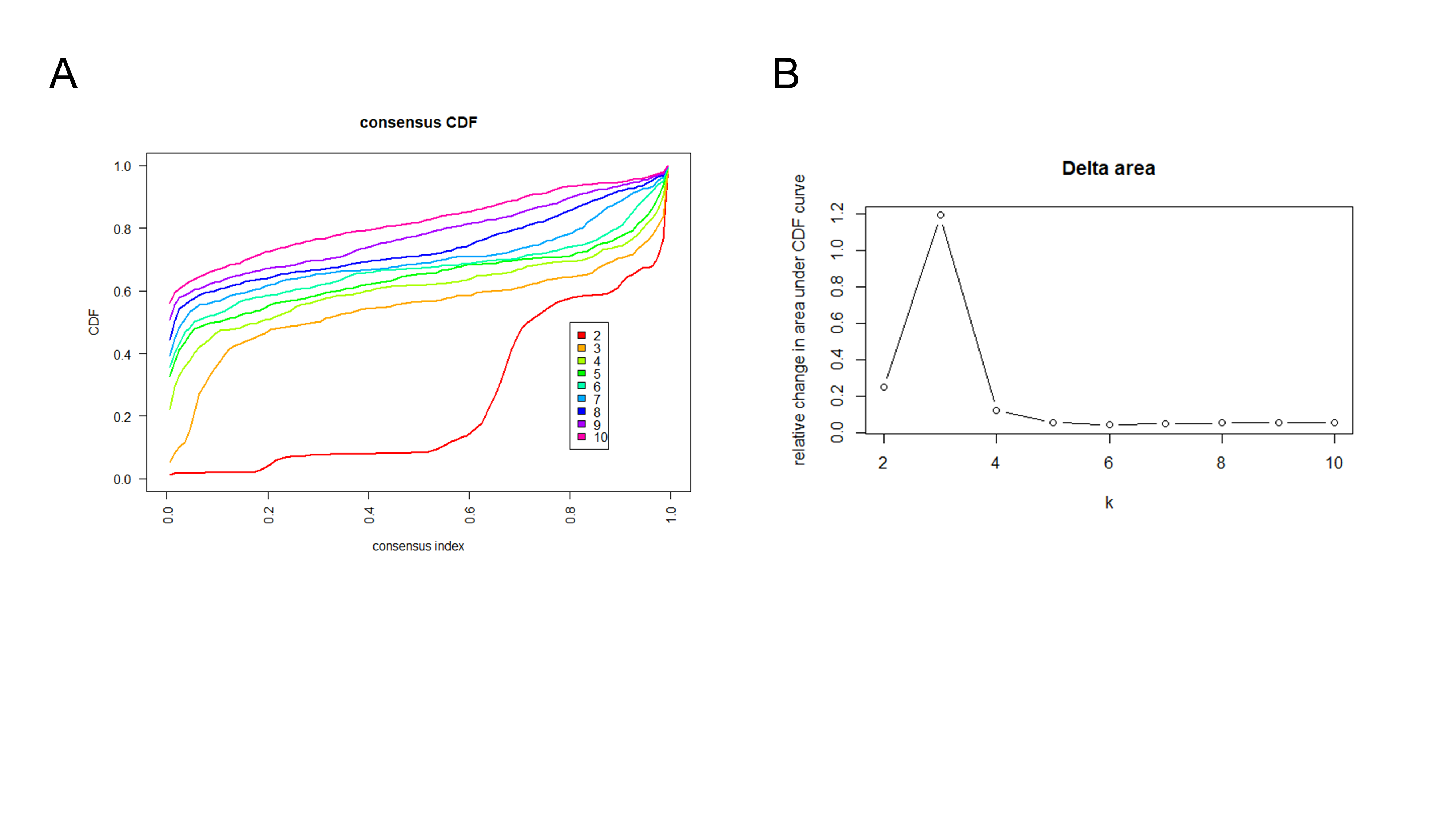

Supplement: Supplementary file 1 [file cancers-14-06207-s001.zip › Supplementary File_for_revision_20221215_v2/FigureS3.tif]
